# Influences of donor and host age on human muscle-derived stem cell-mediated bone regeneration

**DOI:** 10.1186/s13287-018-1066-z

**Published:** 2018-11-21

**Authors:** Xueqin Gao, Aiping Lu, Ying Tang, Johannes Schneppendahl, Andrea B. Liebowitz, Alex C. Scibetta, Elizabeth R. Morris, Haizi Cheng, Charles Huard, Sarah Amra, Bing Wang, Mary A. Hall, Walter R. Lowe, Johnny Huard

**Affiliations:** 1 Department of Orthopaedic Surgery, University of Texas Health Science Center at Houston, Houston, TX 77054 USA; 2 Institute of Molecular Medicine, University of Texas Health Science Center at Houston, Houston, TX 77054 USA; 3 Steadman Philippon Research Institute, Vail, CO 81657 USA; 4 Department of Orthopaedic Surgery, University of Pittsburgh, Pittsburgh, PA 15219 USA

**Keywords:** Bone morphogenetic protein 2 (BMP2), Human muscle-derived stem cells (hMDSCs), Calvarial bone defect, Bone regeneration, Gene therapy, Aging

## Abstract

**Background:**

Human muscle-derived stem cells (hMDSCs) have been shown to regenerate bone efficiently when they were transduced with Lenti-viral bone morphogenetic protein 2 (LBMP2). However, whether the age of hMDSCs and the animal host affect the bone regeneration capacity of hMDSCs and mechanism are unknown which prompted the current study.

**Methods:**

We isolated three gender-matched young and old populations of skeletal muscle stem cells, and tested the influence of cells’ age on in vitro osteogenic differentiation using pellet culture before and after Lenti-BMP2/green fluorescent protein (GFP) transduction. We further investigated effects of the age of hMDSCs and animal host on hMDSC-mediated bone regeneration in a critical-size calvarial bone defect model in vivo. Micro-computer tomography (CT), histology, and immunohistochemistry were used to evaluate osteogenic differentiation and mineralization in vitro and bone regeneration in vivo. Western blot, quantitative polymerase chain reaction (PCR), and oxidative stress assay were performed to detect the effects of age of hMDSCs on cell survival and osteogenic-related genes. Serum insulin-like growth factor 1 (IGF1) and receptor activator of nuclear factor-kappa B ligand (RANKL) were measured with an enzyme-linked immunosorbent assay (ELISA).

**Results:**

We found LBMP2/GFP transduction significantly enhanced osteogenic differentiation of hMDSCs in vitro, regardless of donor age. We also found old were as efficient as young LBMP2/GFP-transduced hMDSCs for regenerating functional bone in young and old mice. These findings correlated with lower phosphorylated p38MAPK expression and similar expression levels of cell survival genes and osteogenic-related genes in old hMDSCs relative to young hMDSCs. Old cells exhibited equivalent resistance to oxidative stress. However, both young and old donor cells regenerated less bone in old than young hosts. Impaired bone regeneration in older hosts was associated with high bone remodeling due to higher serum levels of *RANKL* and lower level of IGF-1.

**Conclusion:**

hMDSC-mediated bone regeneration was not impaired by donor age when hMDSCs were transduced with LBMP2/GFP, but the age of the host adversely affected hMDSC-mediated bone regeneration. Regardless of donor and host age, hMDSCs formed functional bone, suggesting a promising cell resource for bone regeneration.

**Electronic supplementary material:**

The online version of this article (10.1186/s13287-018-1066-z) contains supplementary material, which is available to authorized users.

## Background

Aging has a significant impact on human health and can result in a multitude of problems. Reduced stem cell number or dysfunction is associated with age-related degenerative diseases. Since stem cells have great potential for the treatment of age-related degenerative diseases, such as osteoporosis, fracture non-union, and large bone defects, they are likely candidates for use in elderly individuals to restore loss of tissue homeostasis due to degenerative disease. Therefore, it is important to investigate the effects of donor and host cell aging on stem cell-mediated tissue regeneration.

The effects of age of stem cells on their self-renewal and differentiation capacities have been studied for both non-human animal and human cells; however, discrepancies exist between different studies. Both bone marrow mesenchymal stem cells (BMMSCs) and adipose-derived stem cells (ADSCs) isolated from aged rats have been shown to exhibit increased cell senescence and a trend of increasing p38 and p53 levels with age compared to neonatal and young rats [1]. However, aged BMMSCs and ADSCs were still able to differentiate into Schwann-like cells and maintain neural axonal growth [1]. Older BMMSCs exhibited impaired cell proliferation and multipotent differentiation, while muscle-derived stem cells (MDSCs) and ADSCs were not negatively affected by age [2]. In mice, BMMSCs and ADSCs isolated from old animals were found to display a cell senescence phenotype, but platelet-rich plasma (PRP) treatment was found to reverse the cell senescence and improve osteogenesis and chondrogenesis [3]. Furthermore, human ADSCs isolated from older women have been found to exhibit similar adipogenic capacity, but impaired osteogenic capacity compared to young women [4]. It also has been shown that ADSCs isolated from aged human adipose tissues exhibited decreased proliferation, osteogenesis, and chondrogenesis, and increased cell senescence and adipogenesis [5]. Finally, another study demonstrated that the function of human ADSCs is not affected by age in terms of stem cell differentiation [6]. However, most of these studies have been conducted in vitro.

In fact, there are very few studies which have investigated the effects of host age on bone tissue regeneration. It has been shown that reduced bone graft efficiency in aged animals is related to loss of WNT3A protein, but supplemental liposome-reconstituted WNT3A protein restored the bone formation potential [7]. The age of the recipient animal may also affect the bone regenerative capacity of stem cells; however, to our knowledge, this topic has not been extensively studied.

Human MDSCs (hMDSCs) have been shown to efficiently repair critical-size bone defects when transduced with lentiviral-bone morphogenetic protein 2 (LBMP2) [8]. Also, hMDSCs demonstrated the ability to regenerate functional bone as efficiently as human BMMSCs when transduced to express BMP2 [9]. Despite the abovementioned progress, the effects of the age of the host as well as the age of donor hMDSCs on hMDSC-mediated bone regenerative capacity have not been evaluated and prompted the current study.

## Methods

The use of human tissues was approved by the Institutional Review Board (IRB) of the University of Pittsburgh, and all animal experiments and procedures were approved by the Institutional Animal Care and Use Committee (IACUC) of the University of Pittsburgh. Similarly, research performed at the University of Texas Health Science Center at Houston (UTHealth) was approved by the UTHealth Institutional Biosafety Committee.

The University of Pittsburgh and UTHealth were accredited by the Association For Assessment and Accreditation of Laboratory Animal Care (AAALAC). The use of animals for in vivo study followed the guidelines of the “Basel Declaration” and “ethical guidelines” of the International Council for Laboratory Animal Science (ICLAS).

### Cell isolation

Six populations of hMDSCs were isolated via a modified preplate technique, as previously described [10], from skeletal muscle biopsies purchased from the National Disease Research Interchange (NDRI; Philadelphia, PA). These populations of hMDSCs were from donors of the following ages and grouped into three gender and proliferation rate-matched pairs: pair 1, young 1 (31-year-old female) and old 1 (76-year-old female); pair 2, young 2 (23-year-old male) and old 2 (78-year-old male); and pair 3, young 3 (21-year-old male) and old 3 (80-year-old male). The hMDSCs were grown and maintained in proliferation medium (PM), consisting of high-glucose DMEM (Invitrogen) supplemented with 20% fetal bovine serum (FBS, Invitrogen), 1% chicken embryo extract (Germini), and 1% penicillin/streptomycin (Invitrogen).

### Construction of the LBMP2/GFP vector

A lentiviral vector encoding the human BMP2 gene, under the control of the human cytomegalovirus (CMV) promoter and with a GFP tag separated by an internal ribosome entry site (IRES) from the target gene, was constructed in collaboration with Dr. Bing Wang’s laboratory. The GFP tag facilitates monitoring of transduction efficiency and the use of fluorescence-activated cell sorting (FACS) to select transduced cells. The LBMP2/GFP viral vector was packaged using 293T cells (American Type Culture Collection, ATCC).

### Cell transduction

Human MDSCs were transduced with LBMP2/GFP in the presence of polybrene (8 μg/ml) at passage 8–10 for 16 h. Twenty-four hours after transduction, transduction efficiency was observed using fluorescence microscopy and found to be about 50–60%. Cells were passaged twice after transduction and then subjected to cell sorting using FACS to isolate cells having the GFP tag. After sorting, the cells were expanded in proliferation media. Supernatants were collected from different passages of each population, and BMP2 secretion levels were measured using a BMP2 quantikine ELISA kit (DBP200, R&D Systems).

### In vitro osteogenic differentiation using 3D pellet cultures

The six populations of hMDSCs were subjected to pellet culture before and after LBMP2/GFP transduction, using a previously described protocol [8]. Four replicate pellets were prepared for each population and cultured in osteogenic medium utilizing the same conditions. At 4 weeks after initiating the osteogenic cultures, the pellets were scanned with a microCT (Viva CT 40, Scanco Medical) to detect mineralization using a voxel size of 21 and medium resolution. The mineralized pellet volumes were evaluated using the following parameters: Gauss Sigma 0.8, Gauss support 1.0, and threshold 122. After microCT scanning, the cell pellets were fixed in 4% neutral buffered formaldehyde (NBF; Sigma-Aldrich) for 1 h at room temperature, rinsed one time with phosphate-buffered saline (PBS), and then embedded in NEG 50 freezing medium, snap-frozen in liquid nitrogen, and stored at − 80 °C until they were cryosectioned at 8-μm thickness. Von Kossa staining was performed using an online protocol (http://www.ihcworld.com/_protocols/special_stains/von_kossa.htm) to verify pellet mineralization. Osteocalcin immunohistochemistry using a mouse anti-human osteocalcin primary antibody (MAB1419,1:100, R&D Systems) was also performed, as previously reported [9]. The diaminobenzidine (DAB) color reaction was used to reveal osteogenic differentiation, as indicated by a brown color. Pellet culture experiments were repeated three times for all cell populations.

### Creation of critical-size calvarial bone defects

Critical-size calvarial bone defects were created using our established protocol [11]. Briefly, ICR-SCID mice (Taconic) were anesthetized using 2% isoflurane, an incision was made just off the middle line of the skull on the scalp, and the right parietal bone was exposed. After removal of the periosteum, a 5-mm bone defect was created using a 5-mm diameter trephine (Fine Science Tools). The bone was carefully removed, and the defect area was rinsed with normal saline. The culture-expanded LBMP2/GFP-transduced hMDSCs were resuspended in 20 μl PBS and mixed with 20 μl thrombin immediately before their transplantation into the defect area. Following cell transplantation, 20 μl of fibrin sealant (Tisseel, Baxter) was placed on top of the cells and allowed to solidify for 1–2 min. The wound was closed with sutures, and the mice were allowed to recover in an oxygen chamber while under observation.

### Comparison of bone regeneration in vivo using young and old donor LBMP2/GFP-transduced hMDSCs in young animal hosts

Eight-week-old (young host) male ICR-SCID mice (Taconic) were divided into six groups (*N* = 6). Mice from three of the groups were transplanted with 1.5 × 10^6^ LBMP2/GFP-transduced hMDSCs from the young 1, young 2, or young 3 cell population, respectively. Mice from each of the other three groups were transplanted with 1.5 × 10^6^ LBMP2/GFP-transduced hMDSCs from the old 1, old 2, or old 3 cell population, respectively. MicroCT scans (Viva CT-40, Scanco Medical, Switzerland) were performed at day 1, and 2, 4, and 6 weeks after calvarial defect (post-injury) and donor cell transplantation using a 30-μm voxel size and medium resolution. Bone volume was quantified using Scanco evaluation software according to the guidelines of the American Society of Bone and Mineral Research (ASBMR) [12].

### Comparison of bone regeneration in vivo using young and old donor LBMP2/GFP-transduced hMDSCs in old animal hosts

Nine- to 12-month-old (old host) male ICR-SCID mice (Taconic) were divided into six groups (*N* = 6). Mice from three of the groups were transplanted with 1.5 × 10^6^ LBMP2/GFP-transduced hMDSCs from the young 1, young 2, or young 3 cell population, respectively. Mice from each of the other three groups received 1.5 × 10^6^ LBMP2/GFP-transduced hMDSCs from the old 1, old 2, or old 3 cell population, respectively. MicroCT scanning was performed and bone volume was quantified as above-described.

### Histology

In all cases, skull tissues containing the defect were harvested at 6 weeks post-injury, fixed in 10% NBF for 1 week, and then decalcified using 10% ethylenediaminetetraacetic acid disodium (EDTA-Na_2_) containing 1% sodium hydroxide for 4 weeks. The tissues were harvested immediately following euthanasia, according to approved institutional protocols (see above). Tissues were paraffin-embedded and 5-μm sections were cut. Herovici’s staining was used to identify collagen type I, as previously described [13]. Hematoxylin and eosin (H&E) staining was used to reveal the newly regenerated bone and bone marrow. Tartrate-resistant acid phosphatase (TRAP) staining was performed on the decalcified paraffin sections using a 387A kit (Sigma). TRAP-positive osteoclasts on the bone surface were normalized to bone area (excluding bone marrow area) and expressed as cell number/mm^2^.

GFP immunohistochemistry was then carried out on 5-μm paraffin sections to track the contributions of donor cells to the regenerated bone. After deparaffinization, washing, and blocking with 5% donkey serum in PBS, sections were incubated with rabbit anti-GFP antibody (ab290, Abcam, 1:1000 dilution) overnight. The following day, sections were treated with 0.5% H_2_O_2_ in PBS for 30 min at room temperature, washed in PBS, and incubated with goat anti-rabbit-biotin (BA 1000, Vector Laboratories, 1:200 dilution) for 2 h at room temperature. After three washes, each slide was incubated with ABC reagents (PK 7200, Elite ABC kits, Vector Laboratories) for 2 h at room temperature. Staining with DAB (SK-4100, Vector Laboratories) was used to visualize GFP^+^ cells. Hematoxylin QS (H-3404, Vector Laboratories) counterstaining was performed following the DAB reaction.

### ELISA

Whole blood was collected from each mouse at the time of sacrifice (euthanasia) of young and old animals at 6 weeks post-injury, and serum was isolated and stored at − 80 °C until testing by ELISA. Levels of IGF1 (MG100), RANKL (MTR00), and sclerostin (MSST00) were measured using ELISA kits from R&D Systems.

### Oxidative stress assays

For oxidative stress experiments, 2 × 10^3^ young or old donor hMDSCs were seeded in 24-well plates and cultured at 37 °C with 5% carbon dioxide overnight in proliferation medium. The following day, the proliferation medium was removed and cells were rinsed with PBS one time, and then, oxidative stress medium was added to four wells, each containing 500 or 650 μM H_2_O_2_ and propidium iodide (PI; 2 μg/ml). Only dead cells became intercalated with PI, which results in red fluorescence. The plates were set up in a NIKON live-cell imaging system. Four locations (fields of view) from each well were randomly chosen for image capture. Bright-field and red fluorescence images were taken every 10 min, and the number of dead cells was calculated at 0, 4, 8, 12, 16, 20, and 24 h using ImageJ software. The cell survival rates for young and old hMDSCs were calculated for each time point.

### Quantitative reverse transcription polymerase chain reaction (qRT-PCR)

Three pairs of cell populations of young and old hMDSCs, both non-transduced (passage 10–15) and LBMP2/GFP-transduced (at passages 6–8 after GFP cell sorting), were cultured in proliferation medium and then trypsinized with 0.1% Trypsin-EDTA (Invitrogen) and centrifuged. Subsequently, 2 × 10^5^ hMDSCs from each population were lysed with 1 ml of Trizol (Invitrogen). Total RNA was extracted using the protocols provided by the manufacturer. Reverse transcription was performed using 1 μg of total RNA with an iScript Reverse Transcription Supermix kit for qRT-PCR (Bio-Rad). The cDNA from each hMDSC population was diluted with DNAase/RNAase-free water and stored at − 20 °C for further PCR amplification. PCR primers for the genes encoding human cyclooxygenase 2 (COX2), Sry box 9 (SOX9), runt-related X 2 (RUNX2), osterix (OSX), glutamine peroxidase 1 (GPX1), IGF1, and insulin-like growth factor 2 (IGF2) were designed using Primer 3 [14, 15]. For each cDNA template, qRT-PCR was performed in a 20-μl reaction containing specific primers and SsoAdvanced™ Universal SYBR® Green Supermix (Bio-Rad) using a CFX Connect™ real-time thermal cycler (Bio-Rad). The human gene encoding glyceraldehyde 3-phosphate dehydrogenase (GAPDH) was used as the house-keeping control gene. We used the delta CT value (target gene CT value minus CT of GAPDH) to indicate the gene expression level, as it can reflect both abundances and changes in the genes. Primer information is shown in Additional file 1: Table S1.

### Western blot analysis

Cell lysates of six populations of non-transduced hMDSCS were prepared in radioimmunoprecipitation assay (RIPA) buffer (#9806, Cell Signaling Technology, Inc.; Danvers, MA) supplemented with protease inhibitor (P8340) and phosphatase inhibitors (P5726 and P0044); each inhibitor was diluted 1:100 (Sigma–Aldrich). The protein concentration was quantified using a Pierce™ BCA Protein Assay Kit (#23225, Thermo Scientific). Western blot analyses were performed using mouse anti-pAkt (Product #4051, 1:1000 dilution, Cell Signaling Technology), rabbit anti-phospho-p38MAPK (Thr180/Tyr182, D3F9, XP®, #4511, 1:1000, Cell Signaling Technology), rabbit anti-human CDKN2A/p16INK4a [EPR1473] (ab108349, 1:2000, Abcam), and mouse anti-beta actin (A5441, Sigma, 1:8000 dilution) as primary antibodies. Horseradish peroxidase (HRP)-conjugated rabbit anti-mouse (#31450, Pierce, 1:10,000 dilution) and goat anti-rabbit (#31460, Pierce, 1:10,000 dilution) secondary antibodies were used to detect mouse and rabbit primary antibodies, respectively. Bio-Rad Clarity™ and Clarity Max™ Western ECL-blotting substrates were used to reveal target protein bands. The Bio-Rad Chemidoc Touch system was used to capture digital images. Band densities were quantified using Image Lab software from Bio-Rad and then normalized to that of actin.

### Statistical analysis

One-way analysis of variance (ANOVA) or the Student *t* test was used to analyze and compare quantitative data between young and old donors and young and old hosts. For data with high standard deviations, we used the Wilcoxon rank sum non-parametric test. A value of *P* < 0.05 was considered statistically significant.

## Results

### BMP2 secretion levels and in vitro osteogenic differentiation

**Fig. 1:**
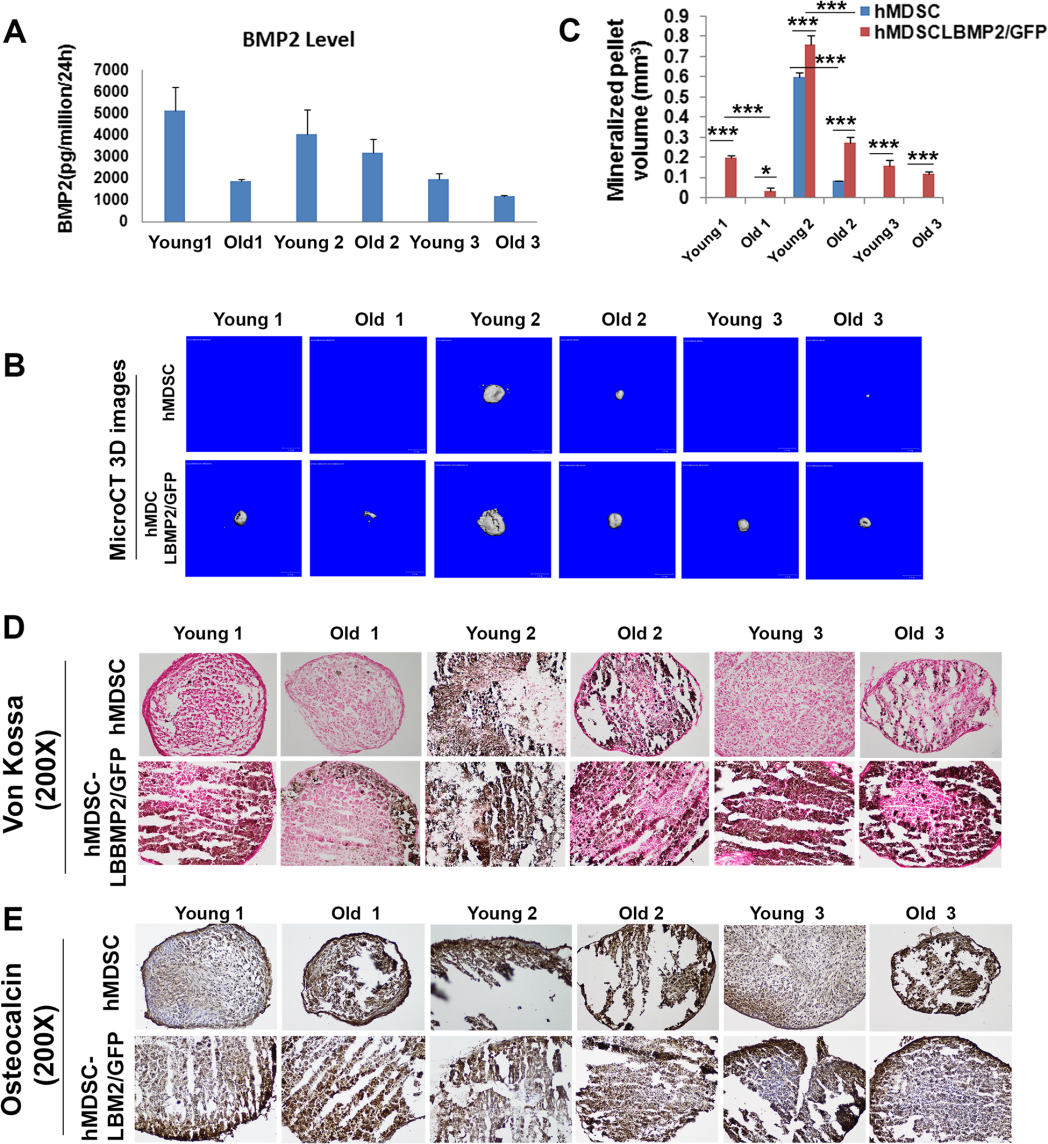
In vitro osteogenesis of young and old donor hMDSCs. **a** BMP2 secretion levels of 6 populations of LBMP2/GFP-transduced cells. **b** MicroCT 3D images of pellet culture for non-transduced and LBMP2/GFP-transduced hMDSCs. LBMP2/GFP-transduced hMDSCs showed larger mineralized pellets in all groups. **c** Quantification of mineralized pellet volume showed significantly higher mineralized pellet volume in all LBMP2/GFP-transduced cells compared to each respective non-transduced hMDSC counterpart. Young donor 1 LBMP2/GFP-transduced cells formed larger pellets than old donor 1 LBMP2-transduced cells. Young donor 2 LBMP2/GFP-transduced hMDSCs also formed larger pellets than old donor 2 cells. No significant difference was found between young donor 3 and old donor 3 LBMP2/GFP-transduced cells. Young donor 2 non-transduced hMDSCs also formed larger mineralized pellets than old donor 2 non-transduced hMDSCs. **d** Von Kossa staining showed that LBMP2/GFP-transduced hMDSCs had more mineralization (as shown in black) than did non-transduced cells. **e** Osteocalcin immunochemistry revealed the osteogenic differentiation of both non-transduced and LBMP2/GFP-transduced hMDSCs in all groups. Note that highly mineralized parts of the cell pellets often peeled away. *P < 0.05, ***P < 0.001

In order to test whether the age of donor hMDSCs affects their osteogenic potential and bone regenerative capacity, we isolated three gender-matched pairs of young and old hMDSCs. We transduced each population of the three young and old hMDSC pairs with LBMP2/green fluorescent protein (LBMP2/GFP) under the same conditions using a multiplicity of infection (MOI) of 8. We measured levels of BMP2 produced by the LBMP2/GFP-transduced cells after sorting via FACS for GFP and subsequent cell culture. The BMP2 secretion levels ranged between 1 and 6 ng/million cells/24 h for young and old cells (Fig. 1a). In vitro pellet culture demonstrated that LBMP2/GFP-transduced hMDSCs appeared to form larger mineralized pellets than did non-transduced cells in all pairs, as shown by micro-computed tomography (microCT) 3D images (Fig. 1b). Quantification of mineralized pellet volume, indeed, showed significantly higher mineralized pellet volume in all LBMP2/GFP-transduced hMDSCs compared to non-transduced hMDSCs, regardless of donor age (Fig. 1c). Von Kossa staining also demonstrated that LBMP2/GFP-transduced hMDSCs had more mineralization than non-transduced hMDSCs, regardless of donor age (Fig. 1d). Osteocalcin immunohistochemistry demonstrated enhanced osteogenic differentiation of LBMP2/GFP-transduced cells in all pairs (Fig. 1e).

### Bone formation mediated by old donor LBMP2/GFP-transduced hMDSCs is not compromised compared to young donor LBMP2/GFP-transduced hMDSCs in young or old animal hosts

**Fig. 2:**
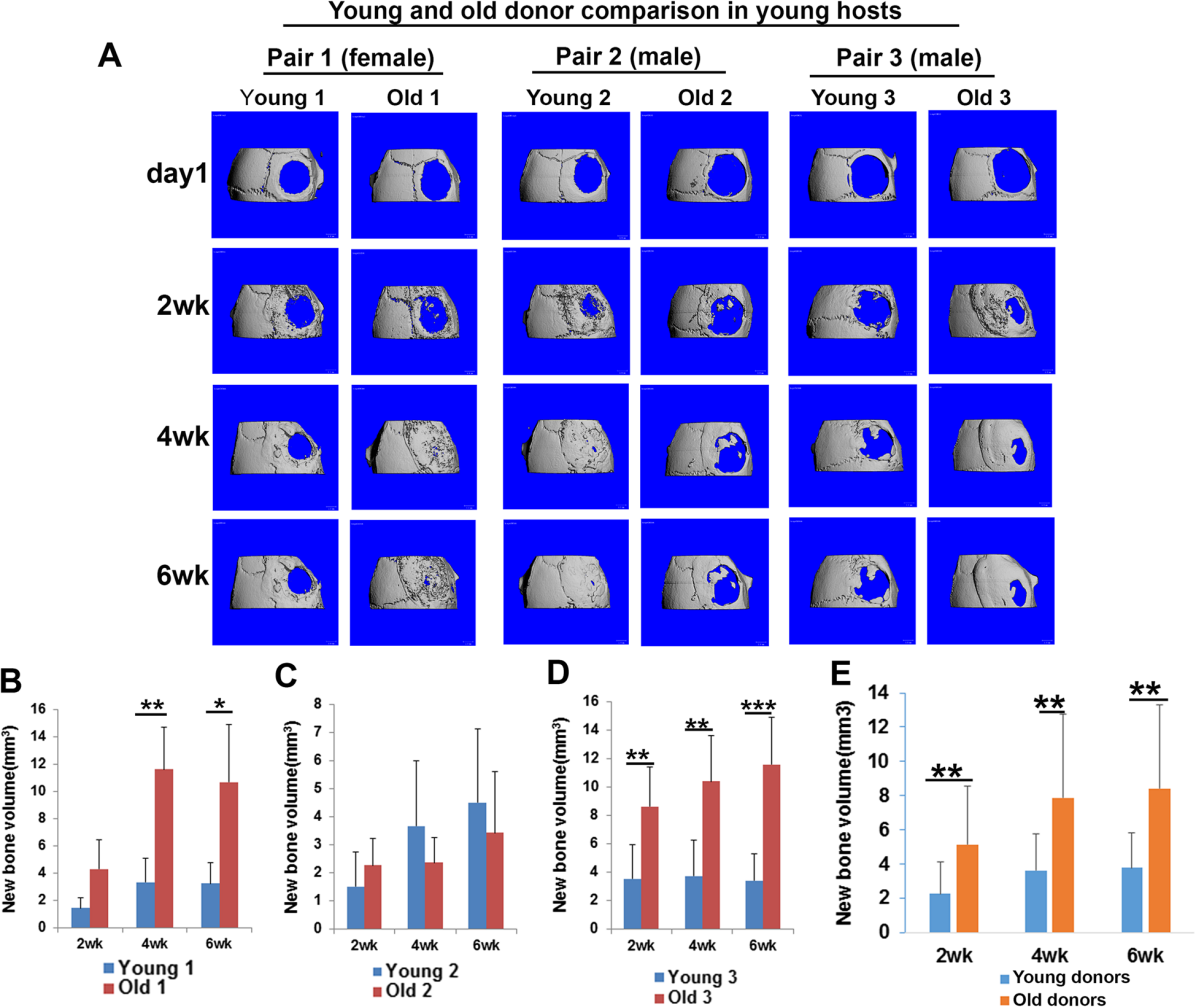
Comparison of bone regeneration mediated by young and old donor LBMP2/GFP-transduced hMDSCs after transplantation in young animal hosts. **a** MicroCT 3D images of skull defect injury and new bone volume quantification using three pairs of young and old donor hMDSCs. **b** New bone volume was significantly higher when using old donor 1 cells compared to using young donor 1 cells at 4 and 6 weeks post-injury. **c** New bone volume was not significantly different between young donor 2 and old donor 2 cells at any time point. **d** New bone volume was significantly higher when using old donor 3 cells compared to using young donor 3 cells. **e** When results for all three old donor cells were combined and compared to combined results for all three young donor cells, new bone volume was significantly higher at 2, 4, and 6 weeks post-injury when old donor cells were transplanted. *P < 0.05, **P < 0.01

**Fig. 3:**
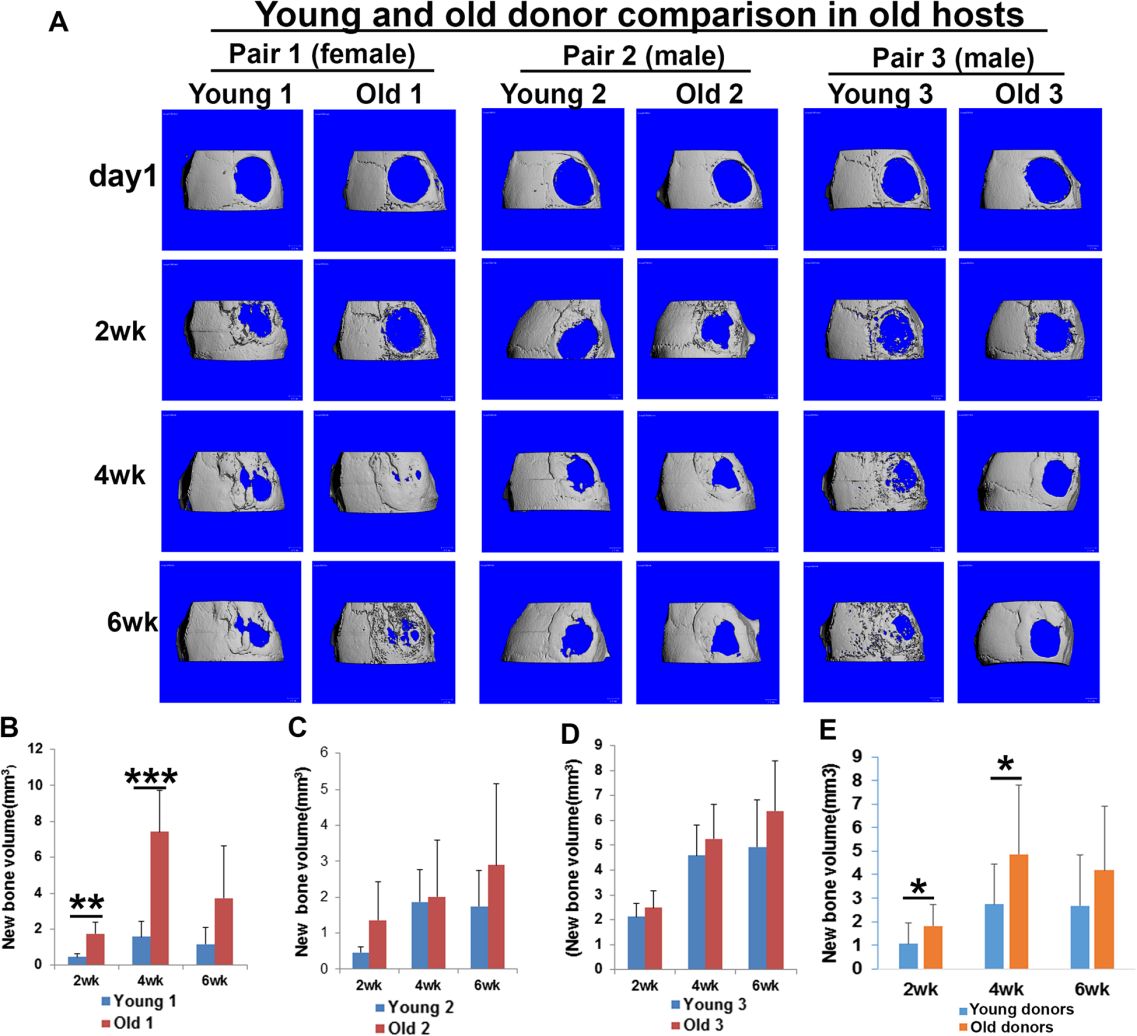
Comparison of bone regeneration mediated by young and old donor LBMP2/GFP-transduced hMDSCs after transplantation in old animal hosts. **a** MicroCT 3D images of skull defect injury and new bone volume quantification using three different young and old donor hMDSCs. **b** New bone volume was significantly higher when using old donor 1 cells compared to young donor 1 cells in old animal hosts at 2 and 4 weeks post-injury. **c**, **d** No significant difference in new bone volume was found between young donor 2 and old donor 2 cells, nor young donor 3 and old donor 3 cells, in old animal hosts at any time point. **e** When results for all three old donor cells were combined and compared to combined results for all three young donor cells, significantly more new bone volume was formed by 2 and 4 weeks post-injury when old donor cells were transplanted. *P < 0.05

In order to investigate whether donor cell age affects bone regeneration, we paired young and old donor cells (pairs 1, 2, and 3) based on the donor’s gender and cell proliferation rate in vitro for comparisons. When we transplanted LBMP2/GFP-transduced hMDSCs in young mouse hosts following critical-size calvarial bone defect injury, we found that bone regenerative capacity using old LBMP2/GFP-transduced hMDSCs was as good as that observed using young LBMP2/GFP-transduced hMDSCs (Fig. 2a). Significantly more new bone was formed by weeks 4 and 6 post-injury in the defect area after we transplanted old donor 1 cells compared to young donor 1 cells in young hosts (Fig. 2b). No significant differences were found between young donor 2 and old donor 2 cells (Fig. 2c). Significantly more new bone volume was regenerated by 2, 4, and 6 weeks post-injury after transplantation of old donor 3 compared to young donor 3 LBMP2/GFP-transduced cells (Fig. 2d). When we compared all three young donor to the three old donor LBMP2/GFP-transduced hMDSCs, we found that old LBMP2/GFP-transduced hMDSCs regenerated significantly more bone than did young LBMP2/GFP-transduced hMDSCs by 2, 4, and 6 weeks post-injury in young hosts (Fig. 2e). Similarly, in old mouse hosts, we found significantly more new bone formation when using old donor 1 cells compared to young donor 1 cells (Fig. 3a, b). No significant differences were found between young donor 2 and old donor 2 or young donor 3 and old donor 3 LBMP2/GFP-transduced cells in old mouse hosts (Fig. 3c, d). When all three young were compared with all three old LBMP2/GFP-transduced hMDSCs, we found that by 2 and 4 weeks post-injury, old LBMP2/GFP-transduced hMDSCs had regenerated significantly more bone than young LBMP2/GFP-transduced hMDSCs (Fig. 3e). No statistically significant difference was found at 6 weeks post-injury in old hosts. These results, together, indicate that bone regenerative capacity of LBMP2/GFP-transduced hMDSCs was not impaired by using older donor cells.

### Both young and old donor hMDSCs regenerate less bone in old hosts than in young hosts

**Fig. 4:**
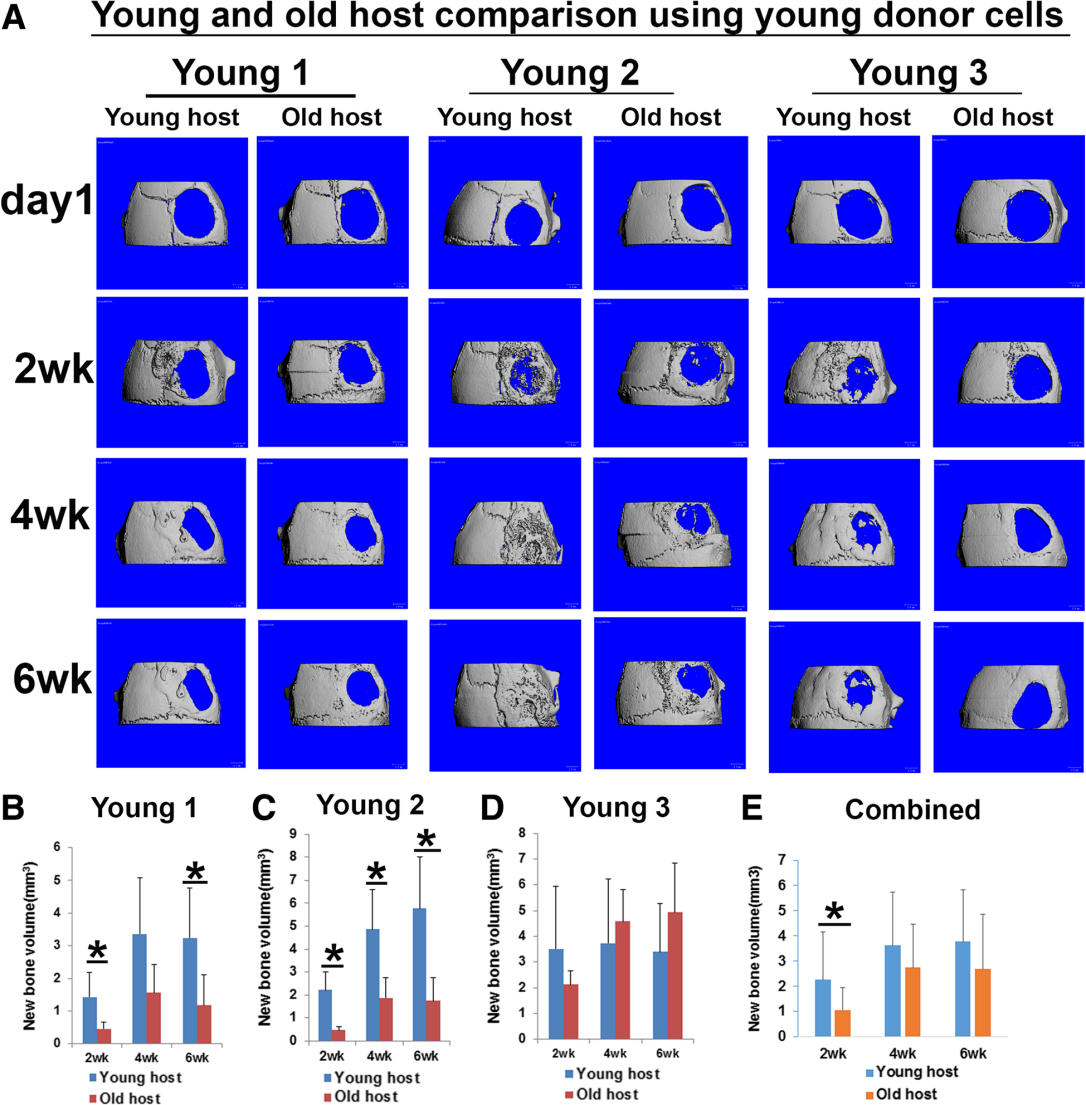
Comparison of bone regeneration in young and old animal hosts after transplantation of young donor LBMP2/GFP-transduced hMDSCs. **a** MicroCT 3D images of skull defect injury and new bone volume quantification using young donor 1–3 cells. **b** New bone volume was significantly lower in old hosts when compared to young hosts at 2 and 6 weeks post-injury after young donor 1 cells were transplanted. **c** New bone volume was significantly lower in old hosts when compared to young hosts at 2, 4, and 6 weeks post-injury after using young donor 2 cells. **d** New bone volume was not significantly different between young and old hosts at any time point when young donor 3 cells were used. **e** When results for all three old hosts were combined and compared to combined results for all three young hosts, significantly less new bone formation was observed in old hosts at 2 weeks post-injury; differences were not significant at 4 or 6 weeks post-injury. *P < 0.05

**Fig. 5:**
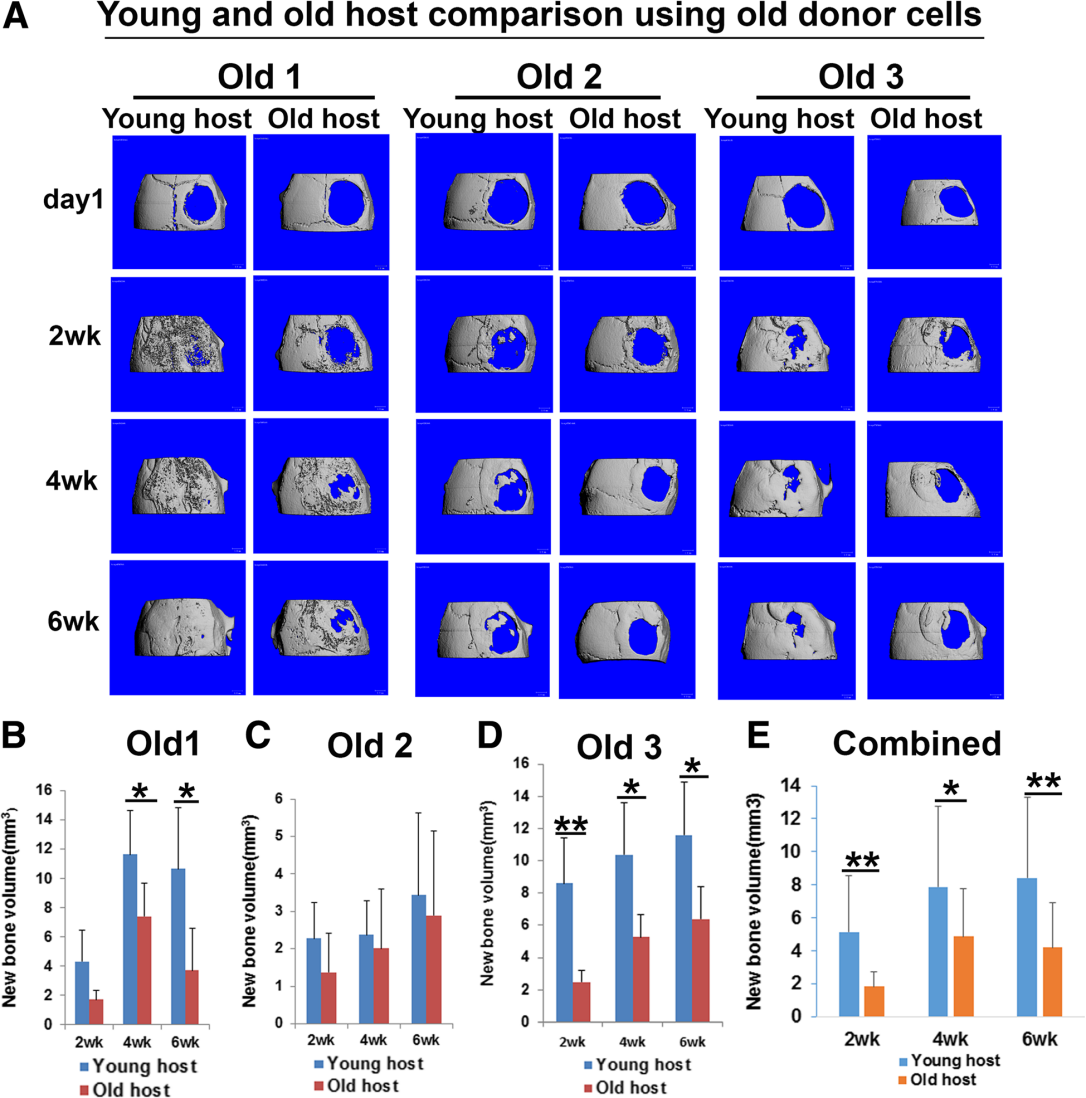
Comparison of bone regeneration in young and old animal hosts after transplantation of old donor LBMP2/GFP-transduced hMDSCs. **a** MicroCT 3D images of skull defect injury and new bone volume quantification using old donor 1–3 cells. **b** New bone volume was significantly lower in old hosts than in young hosts at 4 and 6 weeks post-injury after old donor 1 cells were transplanted. **c** No difference was found for new bone volume in young and old hosts at any time point when old donor 2 cells were used. Of note, the new bone volume regenerated using old donor 2 cells was smaller than that using the other two old donor cell populations (old donor 1 and old donor 3) at respective time points. **d** New bone volume was significantly lower in old hosts compared to young hosts at all time points measured when old donor 3 cells were used. **e** When results for all three old hosts were combined and compared to combined results for all three young hosts, new bone volume was significantly lower in old hosts at 2, 4, and 6 weeks post-injury. *P < 0.05, **P < 0.01

Next, we compared the effect of age of the animal host on bone regeneration mediated by LBMP2/GFP-transduced young and old hMDSCs. We found, when we transplanted young donor 1 and young donor 2 LBMP2/GFP-transduced hMDSCs, significantly lower bone volumes were formed in old mouse hosts than in young mouse hosts (Fig. 4a–c). No significant differences were found between young and old mouse hosts when using young donor 3 cells (Fig. 4d). When we combined results for all three young LBMP2/GFP-transduced cells, significantly less bone was regenerated in old hosts by 2 weeks post-injury when compared to young hosts (Fig. 4e), although no significant differences were found at 4 or 6 weeks post-injury. We also found significantly less new bone in the defect area of old mouse hosts than young mouse hosts when we used old donor 1 (by 4 and 6 weeks post-injury) and old donor 3 (by 2, 4, and 6 weeks post-injury) LBMP2/GFP-transduced hMDSCs (Fig. 5a, b, d). No significant differences were found for new bone volume between young and old mouse hosts when using old donor 2 LBMP2/GFP-transduced hMDSCs at any time point (Fig. 5a, c). When we combined results for all 3 old donor LBMP2/GFP-transduced hMDSCs, we found significantly less regenerated bone at all the time points measured in old animal hosts when compared to young animal hosts (Fig. 5e).

### Histological analyses of bone formation

**Fig. 6:**
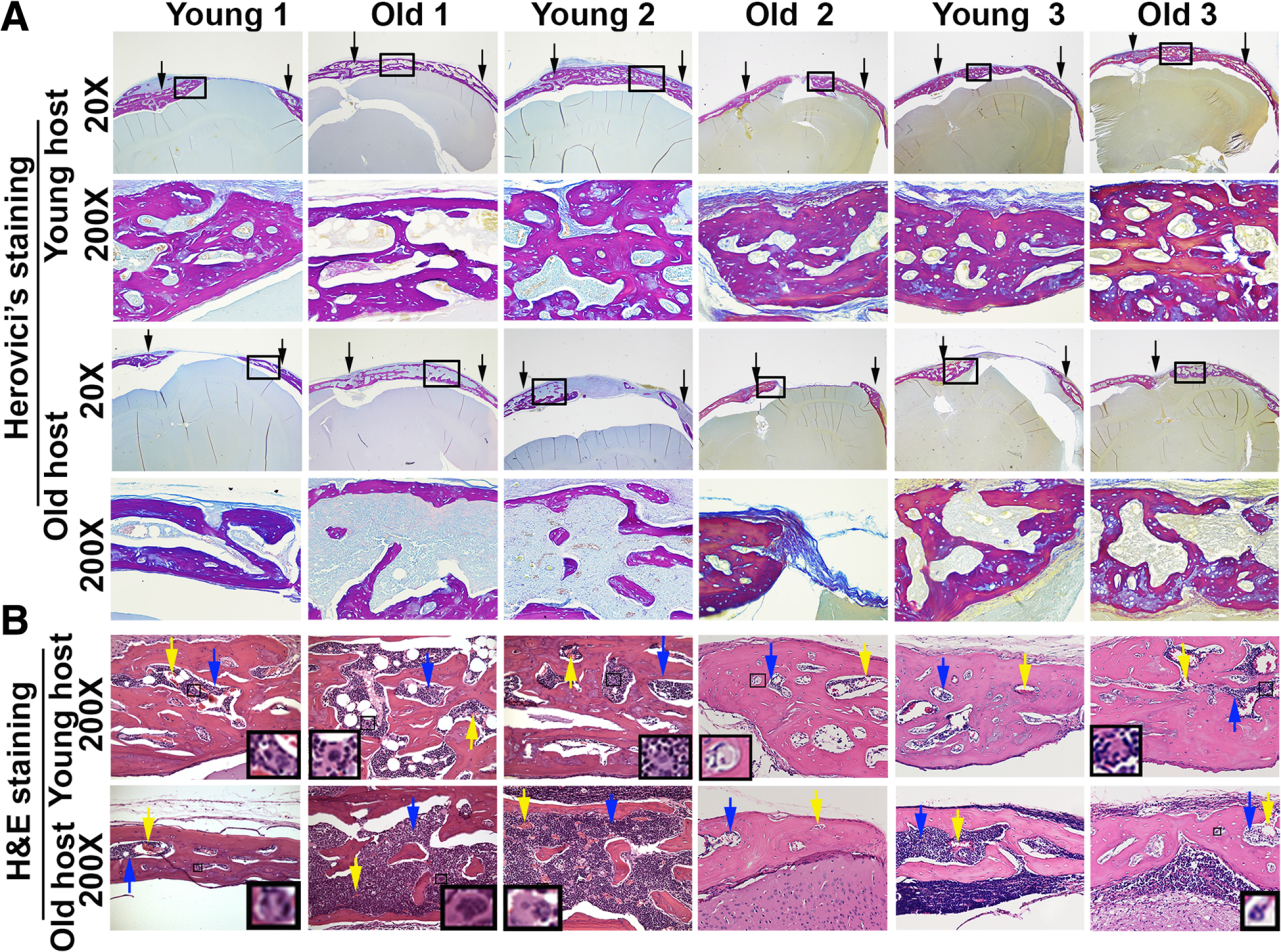
Representative Herovici’s and H&E staining of regenerated bone. **a** Herovici’s staining showed positive bone matrix collagen type I (Col1, stained pink red; bone marrow [BM] light blue and collagen type3 [Col3], blue) in the defect area in all groups. All groups stained positive for Col1, as displayed by the trabecular-like red structure, when young and old donor cells were transplanted into young or old hosts. However, new bone formed in old animal hosts was less dense than in young hosts. The area between two black arrows in each picture shows the defect area (× 200 is the box area from × 20). **b** H&E staining indicated that the new bone in all groups was functional bone, as the defect area observed for all groups showed both bone matrix (black arrows in all pictures that point to the tissues other than bone marrow) and bone marrow, with hematopoietic stem cells as well as blood vessels. Insets are the enlarged black boxes in each picture that indicate megakaryocytes. Yellow arrows indicate red blood cells, and blue arrows indicate myeloid cells

Herovici’s staining showed positive bone matrix collagen type 1 in the defect area in all animal hosts (Fig. 6a; collagen type 1 [Col1] in pink/red, collagen type 3 [Col3] in blue). No differences were observed in bone formation for young and old donor cells when transplanted into young or old hosts. However, new bone that formed in old animal hosts was less dense than that in young hosts (Fig. 6a). Hematoxylin and eosin (H&E) staining results demonstrated formation of functional bone tissue when we transplanted LBMP2/GFP-transduced hMDSCs from young as well as old donors, in both young and old hosts. We found typical bone matrix as well as bone marrow cells in all hosts. We identified three cell lineages in the newly formed bone, which included myeloid cells (blue arrows), megakaryocytes (black boxes and insets), and red blood cells (yellow arrows), as well as blood vessels (Fig. 6b).

### Donor cell contributions to bone formation in young and old hosts

**Fig. 7:**
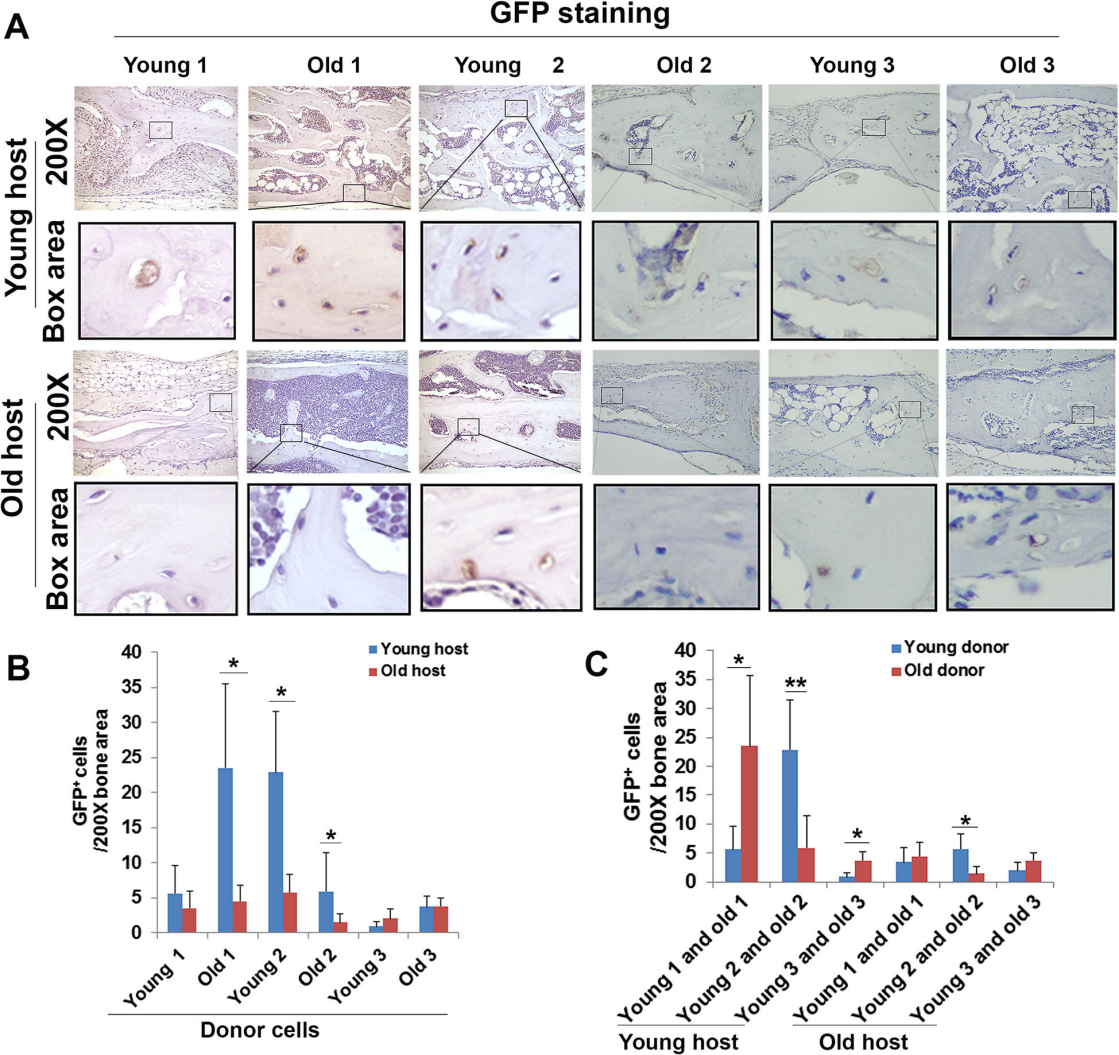
GFP staining for tracing donor cells in the regenerated bone. **a** We found GFP-positive cells in all donor cell groups in young and old animal hosts. The boxed area is an enlarged image from each original image. **b** Quantification of GFP-positive cells in young and old hosts. We found that there were fewer GFP-positive cells in old hosts when compared to young hosts when old donor 1, young donor 2, and old donor 2 cells were used (*P < 0.05). There were no significant differences between young and old hosts when using the other donor cells. **c** Comparison of GFP-positive cells of young and old donor cells. We found differences in the number of GFP-positive cells between young and old donor cells in both young and old hosts. When we transplanted cells in young hosts, we found more GFP-positive cells when using old donor 1 cells compared to using young donor 1 cells. We found the same results when we compared old donor 3 cells and young donor 3 cells. However, the opposite was found when comparing old donor 2 to young donor 2 cells. In old hosts, we found fewer GFP-positive cells when using old donor 2 cells compared to young donor 2 cells. No differences were found when we compared young donor 1 and old donor 1 or young donor 3 and old donor 3 in old hosts. *P < 0.05, **P < 0.01

We performed GFP immunohistochemistry of 5-μm sections of each skull defect (at 6 weeks post-injury) using anti-GFP antibody to trace donor cells to determine their contribution to bone regeneration with respect to age of the animal host. For young versus old hosts, significantly more GFP-positive (GFP^+^) cells were observed when three of the six populations of donor LBMP2/GFP-transduced hMDSCs (old 1, young 2, and old 2 cells) were transplanted into young animal hosts compared to old animal hosts (Fig. 7a, b). No significant differences were found for GFP^+^ cell numbers when using the other donor cell populations (young 1, young 3, and old 3) in young versus old hosts. We also compared GFP^+^ young versus old donor cell numbers in both the young and old hosts. In young hosts, significantly more GFP^+^ old compared to young donor cells were quantified for two of the three donor pairs (pair 1 and 3), while significantly more GFP^+^ young compared to old donor cells were quantified for the third pair (pair 2, Fig. 7c). In old hosts, significantly more GFP^+^ young compared to old donor cells were quantified for one of the three pairs of donors (pair 2), while no significant differences were found for pair 1 or 3 (Fig. 7c). These results indicate that the contributions of donor LBMP2/GFP-transduced hMDSCs to new bone formation varied by host age (i.e., more GFP^+^ cells were found in young hosts). However, no consistent differences were observed for contributions of young versus old donor cells, suggesting the donor cells’ contributions to the new bone were not affected by the age of the donor (Fig. 7c).

### Bone remodeling analysis by tartrate-resistant acid phosphatase (TRAP) staining

**Fig. 8:**
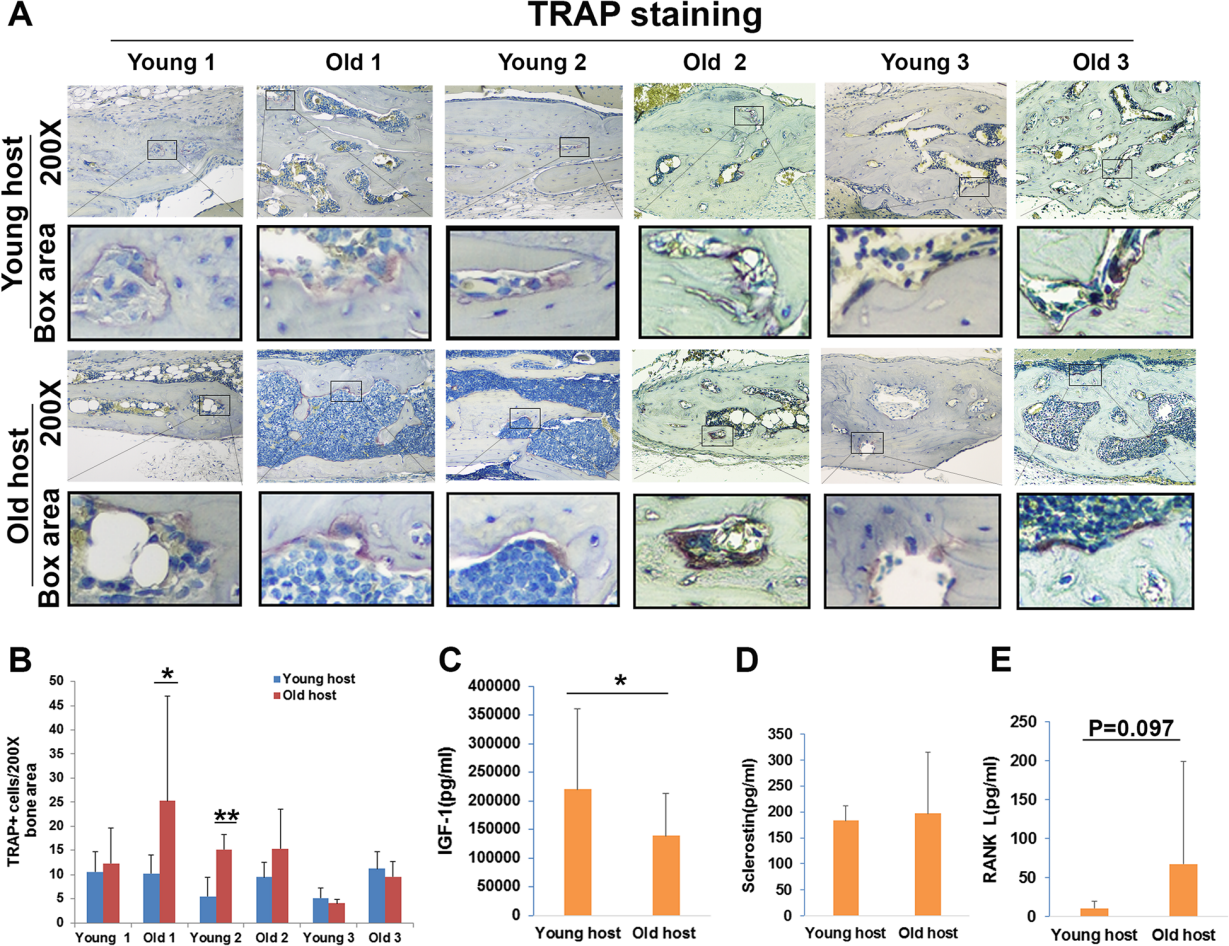
TRAP staining to reveal bone remodeling. **a** TRAP staining for each donor group in young and old host mice. The boxed area is enlarged from each specified respective small box in the original images (× 200). **b** Quantification of TRAP-positive cells in the regenerated bone area. We found significantly more TRAP-positive cells in old hosts when we used old donor 1 and young donor 2 LBMP2/GFP-transduced hMDSCs compared to TRAP-positive cells found in young hosts. No statistically significant differences were found between young and old hosts when using other donor cells. **c** Significantly lower levels of serum IGF1 were found in old hosts compared to young hosts. **d** No significant differences were found for serum sclerostin levels in young and old hosts. **e** A trend of increased serum RANKL levels was found in old hosts compared to young hosts, but differences were not found to be significant. *P < 0.05, **P < 0.01

We performed TRAP (osteoclast marker) staining to reveal bone remodeling. TRAP-positive (violet red) cells were found in every mouse host, indicating newly formed bone underwent remodeling (Fig. 8a). Quantification of TRAP-positive cells in the regenerated bone area demonstrated more TRAP-positive cells in old hosts compared to young hosts when old donor 1 and young donor 2 cells were transplanted (Fig. 8b). No significant differences were observed between young and old hosts when the other donor cells were used.

### Serum levels of circulating factors measured by enzyme-linked immunosorbent assay (ELISA)

To investigate whether systemic factors affect hMDSC-mediated bone regeneration in young and old animal hosts, we performed ELISAs to measure serum levels of three different factors related to bone formation and remodeling, including insulin-like growth factor I (IGF1), sclerostin, and receptor activator of nuclear factor kappa B ligand (RANKL). Serum IGF1 levels were significantly lower in old animal hosts when compared to young hosts (Fig. 8c). No significant differences were observed for serum sclerostin levels between young and old hosts (Fig. 8d). Serum levels of RANKL, a key factor for promoting osteoclastogenesis, appeared higher in old relative to young hosts, although the differences were not found to be statistically significant (Fig. 8e; *P* = 0.097).

### Old hMDSCs are not compromised in terms of cell survival under oxidative stress

We performed oxidative stress experiments to investigate donor cell survival using two different concentrations (500 and 650 μM) of hydrogen peroxide (H_2_O_2_) in culture for 24 h. We found no significant differences between young and old hMDSCs in the three gender-matched pairs of donor hMDSCs (Additional file 1: Figure S1A-F). Of note, we observed high survival rates for all hMDSCs cultured in H_2_O_2_, with most of the cell populations exhibiting > 50% survival at 24 h. This observation suggests that both young and old hMDSCs are resistant to oxidative stress.

### Comparison of protein levels and gene expression in young and old hMDSCs

**Fig. 9:**
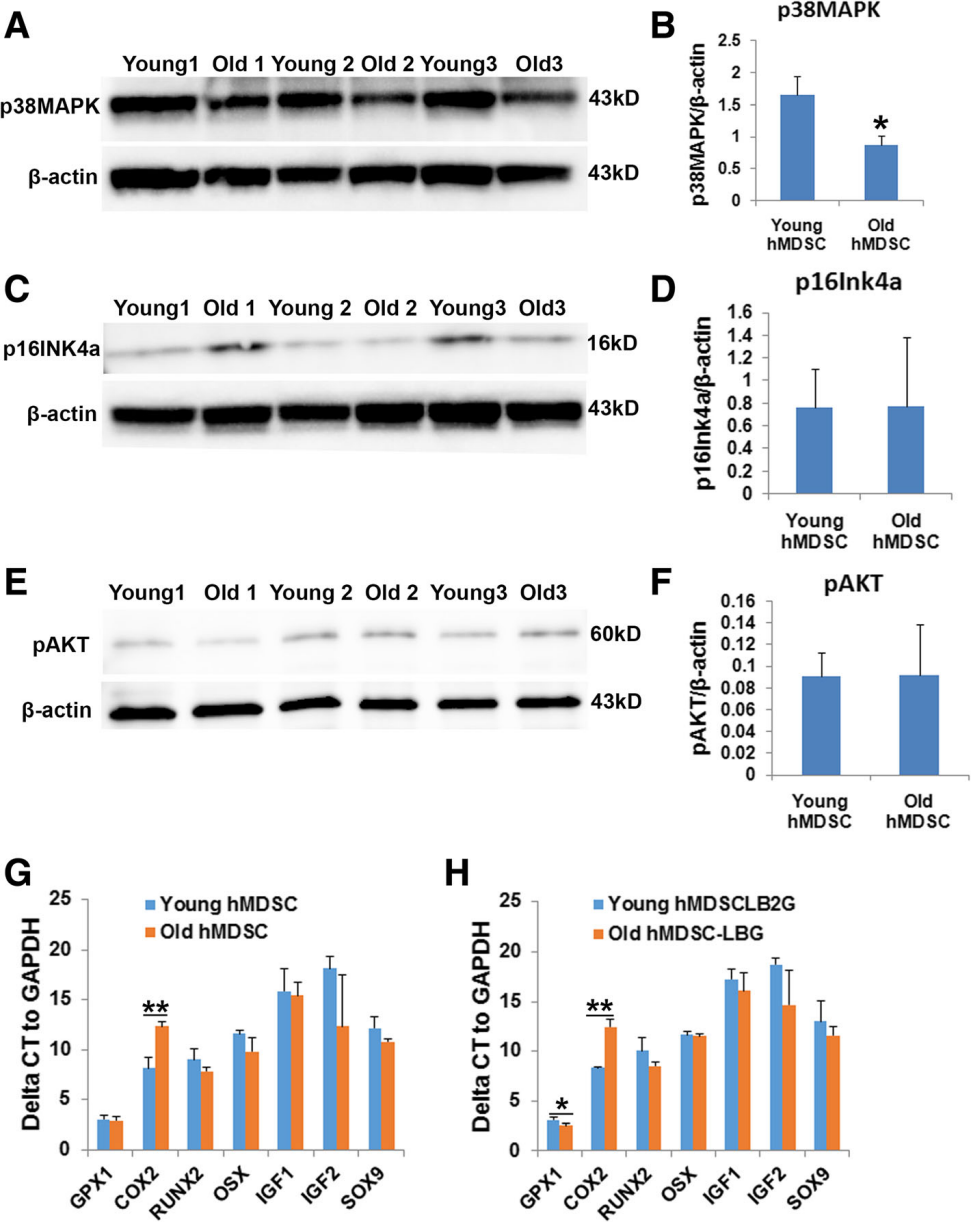
Western blot and qRT-PCR analysis. **a**, **b** Western blot of phosphorylated p38MAPK (pp38MAPK) levels. Old hMDSCs expressed significantly less pp38MAPK compared to young hMDSCs. **c**, **d** Western blot images and quantification revealed no significant differences for the expression of p16IN4a (senescence marker) between young and old hMDSCs. **e**, **f** Western blot images and quantification of pAKT (cell survival marker). No significant differences were observed between young and old hMDSCs. **g** Quantitative RT-PCR (qRT-PCR) analysis of mRNA expression of young and old hMDSCs. COX2 expression was significantly lower (high delta CT) in old hMDSCs than in young hMDSCs. **h** qRT-PCR analysis of young and old LBMP2/GFP-transduced hMDSCs. GPX1 expression was increased, while COX2 expression was decreased in old compared to young LBMP2/GFP-transduced cells. *P < 0.05. **P < 0.01

We observed significantly lower levels of phosphorylated p38 mitogen-activated protein kinase (pp38MAPK) in old hMDSCs compared to young hMDSCs (Fig. 9a, b). No significant differences in protein levels in young versus old hMDSCs were seen for the cell cycle inhibitor (senescence marker) p16INK4a (Fig. 9c, d). Furthermore, no differences in protein levels were observed for the cell survival marker pAKT between young and old hMDSCs (Fig. 9e, f). Results from real-time quantitative reverse transcription polymerase chain reaction (qRT-PCR) for non-transduced young and old hMDSCs indicated that runt-related transcription factor 2 (RUNX2), osterix (OSX), transcription factor SOX9, IGF1, insulin-like growth factor 2 (IGF2), and glutathione peroxidase 1 (GPX1) mRNA levels were similar for young and old hMDSCs (Fig. 9g). The level of cyclooxygenase 2 (COX2) mRNA was significantly lower in old hMDSCs compared to young hMDSCs (Fig. 9g). Results from qRT-PCR for LBMP2/GFP-transduced hMDSCs demonstrated no differences for the RUNX2, OSX, SOX9, IGF1, and IGF2 mRNA levels in old compared to young LBMP2/GFP-transduced hMDSCs (Fig. 9h). GPX1 expression levels were significantly higher in old compared to young LBMP2/GFP-transduced hMDSCs (Fig. 9h). Similar to the trend observed in non-transduced cells, COX2 mRNA was significantly lower in old LBMP2/GFP-transduced hMDSCs compared to young LBMP2/GFP-transduced cells (Fig. 9h).

## Discussion

In this study, we investigated the roles of donor cell age and host age on hMDSC-mediated osteogenesis and bone regeneration in vivo. Our results indicate that both young and old hMDSCs underwent osteogenesis in vitro as evidenced by microCT, Von Kossa staining, and osteocalcin staining. In two gender-matched pairs, old hMDSCs exhibited less osteogenic differentiation than young hMDSCs after LBMP2/GFP transduction; the third pair showed no differences. Conversely, in vivo, we found that old donor cells were able to regenerate new bone in the critical-size defect model in both young and old animal hosts as efficiently as young hMDSCs. However, bone regenerative capacity was reduced in old animal hosts compared to young animal hosts when using either young or old hMDSC populations. Although bone formation was lower in old animal hosts, all the cells formed functional bone in both young and old animal hosts. This finding is very meaningful clinically as stem cells are most likely to be used in older individuals. Our results support the feasibility of using hMDSCs in bone regeneration applications regardless of the age of the donor stem cells. It is generally accepted that aging causes a reduction in the number of tissue stem cells and cell senescence, thereby resulting in loss of tissue homeostasis. However, very few studies have investigated the effects of donor age on stem cell-mediated bone regeneration [1–6]. Thus far, no studies have been reported on the effects of both the age of donor hMDSCs and animal host age on hMDSC-mediated bone regeneration.

In this study, we compared three pairs of gender-matched young and old cells with respect to their in vitro osteogenic capacities, and found that young and old hMDSCs exhibited no differences in osteogenic potentials for two of the three pairs (pair 2 was the exception). However, the young hMDSCs from pair 2 were growing very rapidly when compared to the other cells. The higher mineralized pellet size observed with that cell population may be the result of more rapid proliferation and an increased number of cells during initial osteogenic differentiation, thus resulting in bigger mineralized pellets. LBMP2/GFP transduction significantly enhanced the osteogenic potential of both young and old hMDSCs, although the BMP2 secretion levels by the transduced cells varied somewhat. We did observe that old cells exhibited less osteogenic differentiation than young cells in two pairs; however, this was not observed in the third pair. These observations indicate that hMDSC function is not consistently impaired by age. Indeed, our results are similar to those of a previous study in rabbits, which concluded that MDSC function did not decline with age [2]. The effect of age on self-renewal and differentiation of other human stem cells has been reported [5]. It has been shown that human ADSCs exhibit decreased proliferation, osteogenesis, and chondrogenesis and increased cell senescence and adipogenesis with age [5, 16]. The function of bone marrow MSCs isolated from aged macaques also has been shown to exhibit decline during aging [17]. Our study showed that hMDSCs’ osteogenic differentiation capacities did not decline with age; therefore, we believe hMDSCs are better cell options for bone regeneration, especially in older populations of patients.

Despite the differences in the in vitro osteogenic differentiation of hMDSCs, in vivo induction of new bone regeneration was not impaired after transplantation of old LBMP2/GFP-transduced hMDSCs into critical-size defects compared to transplantation of gender-matched young LBMP2/GFP-transduced hMDSC in both young and old animal hosts. This finding could not be explained by a difference in BMP2 secretion levels, as the mean BMP2 secretion level was lower in old LBMP2/GFP-transduced hMDSCs than in young hMDSCs. Rather, these results imply that the bone regenerative capacity of hMDSCs was not negatively affected by donor cell age after LBMP2/GFP transduction.

Additionally, in vitro oxidative stress testing showed that old cells exhibit oxidative resistance abilities similar to those of young hMDSCs. This finding was also supported by our Western blot analysis, which showed that old cells express similar levels of p16INK4a (cell cycle inhibitor) and pAKT (cell survival marker) when compared with young cells. The protein p16INK4a is a generally accepted molecular marker for senescence and aging [18, 19]. It has been shown that, in the absence of p16INK4a, repopulating defects and apoptosis of hematopoietic stem cells (HSCs) are mitigated, improving the stress tolerance of cells and the survival of animals in successive transplants in a stem cell-autonomous tissue regeneration model [20]. It also has been shown that p16INK4a negatively correlates with population doubling time of human BM MSCs, and its expression is absent in Ki67-positive cells and present in senescence-associated beta-galactosidase-positive cells. Suppression of p16INK4a has been shown to reduce the number of senescent cells and increase cell proliferation of bone marrow MSCs [21]. Furthermore, we found that old hMDSCs express significantly less p38MAPK than do young hMDSCs. It is known that p38MAPK is activated by diverse senescence-associated secretory phenotypes (SASPs) and mainly through the induction of p65 transcriptional activity [22]. It has also been shown that inhibition of p38α/β in murine muscle stem cells (MuSCs) can increase their self-renewal capacity in vitro and aid muscle regeneration in aged mice [23]. Therefore, the decreased pp38MAPK levels in old hMDSCs in our study may have contributed to the high survival of hMDSCs after transplantation, allowing them to regenerate similar or even greater amounts of new bone in both young and old hosts in vivo, compared to young cells.

Importantly, we found that the age of the animal host did affect hMDSC-mediated bone regeneration. We found both young and old donor LBMP2/GFP-transduced hMDSCs regenerated less bone in old animal hosts when compared to young animal hosts. Additionally, our findings showing more TRAP^+^ osteoclasts in the new bone area in old animal hosts may be explained by the relatively higher serum RANKL levels in old animal hosts, which contribute to higher bone remodeling activity. It has been shown that high levels of serum RANKL are associated with lower bone mass in children [24].

Furthermore, we also found that serum IGF1 levels were significantly lower in old animal hosts than young animal hosts. IGF1 is a well-known bone growth factor. IGF1 in bone matrix maintains bone mass via activation of the mechanistic target of rapamycin (mTOR) in mesenchymal cells [25, 26]. The IGF1/IGF1 receptor (IGF1R) axis is also related to bone growth by regulation of OSX and matrix metalloproteinase 13 (MMP13) [27]. Knockout of IGF1R, specifically in osteoblasts, has been shown to impair endochondral bone formation during fracture healing [28]. IGF1 also promotes osteogenic differentiation of dental pulp stem cells [29]. Osteocyte-derived IGF1 is essential for determination of mechanosensitivity [30]. However, conditional knockout of IGF1 in osteocytes, surprisingly, has been shown to accelerate bone fracture healing [31]. Therefore, we believe that downregulation of serum IGF1 in old animal hosts may also contribute to impaired bone regeneration in old hosts regardless of donor cell age.

Few studies have investigated the role of animal host age in bone regeneration. It has been reported that BMMSCs and cortical bone BMP2 levels decrease during aging [32]; however, when the cells were transduced with adeno-BMP2, no differences were observed in osteogenic differentiation of the BMP2-modified BMMSCs among different ages [32]. BMP2-modified BMMSCs were found to regenerate bone in a segmental femur defect in old rats (21 months), whereas non-transduced BMMSCs did not [32]. An earlier study by Quarto et al. showed that adult stromal cells not treated with dexamethasone and implanted subcutaneously in recipient rats exhibited about 10-fold greater bone formation compared to cells from aged rats. On the contrary, dexamethasone-treated BMMSCs from adult and old rats could form significant new bone regardless of donor and recipient ages [33]. These studies, together with our findings, indicate BMP2 plays important roles and may help overcome aging defects in bone regeneration via stem cells.

We also found that old hMDSCs expressed similar levels of RUNX2, OSX, SOX9, IGF1, and IGF2 compared to young hMDSCs. After LBMP2/GFP transduction, the expression levels of these genes were also similar and no significant differences were found between young and old hMDSCs, which may explain why the bone regenerative capacity of old hMDSCs in vivo was not compromised. Notably, COX2 expression was lower in old hMDSCs than in young hMDSCs for both untransduced and LBMP2/GFP-transduced hMDSCs. This finding is consistent with a previous study which showed that a decrease in COX2 in aged mice is associated with delayed fracture healing in the mice [34]. Our previous studies have shown that COX2 knockout murine MDSCs have impaired bone regeneration [35]. The finding that decreased COX2 in old hMDSCs did not significantly affect bone regeneration of old hMDSCs may be due to the fact that COX2 levels in old hMDSCs are still sufficient, and BMP2 can overcome the effects of COX2 downregulation to facilitate bone regeneration.

Lastly, our results also demonstrate that, although bone regeneration mediated by hMDSCs was impaired in old animal hosts, significant amounts of new bone formation were still seen in the defect area in old animal hosts (the defect coverage had no difference, data not shown). The newly formed bone was found to be functional, as shown by both Herovici’s staining and H&E staining. Therefore, the relative degree of impaired bone regeneration observed in old animal hosts should not hinder the application of hMDSCs for bone regeneration, and perhaps, additional time is needed to repair bone in old animals when compared to young hosts.

## Conclusion

Taken together, our study reveals that old donor hMDSCs are as efficient as young donor hMDSCs for regenerating bone in young and old mice. The maintenance of the bone regenerative capacity of old LBMP2/GFP-transduced hMDSCs may be associated with lower levels of ppMAPK38, high levels of GPX 1(after LBMP2/GFP transduction), the ability to express similar levels of osteogenesis-related genes, and equivalent capacity to resist oxidative stress compared to young cells. The relative decrease in new bone formation in old hosts may be attributed to faster bone remodeling due to circulating factors in the serum of young hosts. LBMP2/GFP transduction may overcome the age-related decline in bone regeneration mediated by hMDSCs, as new functional bone was formed in the defect area regardless of donor cell and host age. Whether BMP2 or other BMPs regulate cell cycle activity in aging hMDSCs and, therefore, rescue aging-associated defects in their bone regenerative capacity warrants further investigation. Therefore, hMDSCs are very promising as a cell source for promoting bone regeneration, regardless of donor and host age.

## Additional file

Additional file 1: **Figure S1.** Young and old hMDSCs exhibit similar oxidative stress resistance. Young and old hMDSCs were cultivated in media containing 500 and 650 μM H_2_O_2_ for 24 h in the presence of PI (which labels dead cells) and imaged using a live imaging system. The number of surviving cells and cell survival rate was quantified. (A and B) Comparison of cell survival of the young 1 and old 1 donor cells at 500 μM and 650 μM H_2_O_2_ respectively. (C and D) Comparison of cell survival of the young 2 and old 2 donor cells at 500 and 650 μM H_2_O_2_ respectively. (E and F) Comparison of cell survival of the young 3 and old 3 donor cells at 500 μM and 650 μM H_2_O_2_ respectively. No differences were observed at either concentration for young and old donors. Notably, all hMDSCs were highly resistant to oxidative stress using H_2_O_2_, with survival rates at 650 μM being > 50% for most of the cell populations tested. **Table S1.** primers information. (DOCX 296 kb)

## Abbreviations

ADSCs: Adipose-derived stem cells
ANOVA: One-way analysis of variance
ASBMR: American Society of Bone and Mineral Research
ATCC: American Type Culture Collection
BMMSCs: Bone marrow mesenchymal stem cells
cDNA: Complementary deoxyribonucleic acid
CMV: Cytomegalovirus
COX2: Cyclooxygenase 2
CT: Cycle of threshold line in quantitative PCR.
DAB: Diaminobenzidine
DMEM: Dulbecco’s modified Eagle medium
EDTA-Na_2_: Ethylenediaminetetraacetic acid disodium
FACS: Fluorescence-activated cell sorter
FBS: Fetal bovine serum
GAPDH: Glyceraldehyde 3-phosphate dehydrogenase
GPX1: Glutamine peroxidase 1
hMDSCs: Human muscle-derived stem cells
HRP: Horseradish peroxidase
HSCs: Hematopoietic stem cells
IACUC: Institutional Animal Care and Use Committee
IGF1: Insulin-like growth factor 1
IGF2: Insulin-like growth factor 2
IRB: Institutional Review Board
IRES: Internal ribosome entry site
LBMP2: Lenti-viral bone morphogenetic protein 2
LBMP2/GFP: Lenti-viral bone morphogenetic protein 2/Green fluorescent protein
MicroCT: Micro-computed tomography
MMP13: Matrix metalloproteinase 13
MOI: Multiplicity of infection
NDRI: National Disease Research Interchange,
OSX: Osterix
P16: CDKN2A/p16INK4a, cell cycle inhibitor
p38MAPK: p38 mitogen-activated protein kinase
P53: Tumor suppressor protein
PBS: Phosphate-buffered saline
PI: Propidium iodide
PM: Proliferation medium
PRP: Platelet-rich plasma
qRT-PCR: Quantitative reverse transcription polymerase chain reaction
RANKL: Receptor activator of nuclear factor-kappaB ligand
RIPA: Radioimmunoprecipitation assay
RNA: Ribonucleic acid
RUNX2: Runt-related X 2
SASPs: Senescence-associated secretory phenotypes
SOX9: Sry box 9
TRAP: Tartrate-resistant acid phosphatase
WNT3A: A member of the WNT family that activates beta-catenin
