# Supplementary material for: Influences of donor and host age on human muscle-derived stem cell-mediated bone regeneration
Source: Stem Cell Res Ther. 2018 Nov 21;9:316. doi: 10.1186/s13287-018-1066-z (PMC6249775; doi:10.1186/s13287-018-1066-z)
Supplement: Supplementary file 1 — Figure S1. Young and old hMDSCs exhibit similar oxidative stress resistance. Young and old hMDSCs were cultivated in media containing 500 and 650 μM H2O2 for 24 h in the presence of PI (which labels dead cells) and imaged using a live imaging system. The number of surviving cells and cell survival rate was quantified. (A and B) Comparison of cell survival of the young 1 and old 1 donor cells at 500 μM and 650 μM H2O2 respectively. (C and D) Comparison of cell survival of the young 2 and old 2 donor cells at 500 and 650 μM H2O2 respectively. (E and F) Comparison of cell survival of the young 3 and old 3 donor cells at 500 μM and 650 μM H2O2 respectively. No differences were observed at either concentration for young and old donors. Notably, all hMDSCs were highly resistant to oxidative stress using H2O2, with survival rates at 650 μM being > 50% for most of the cell populations tested. Table S1. primers information. (DOCX 296 kb) [file 13287_2018_1066_MOESM1_ESM.docx]

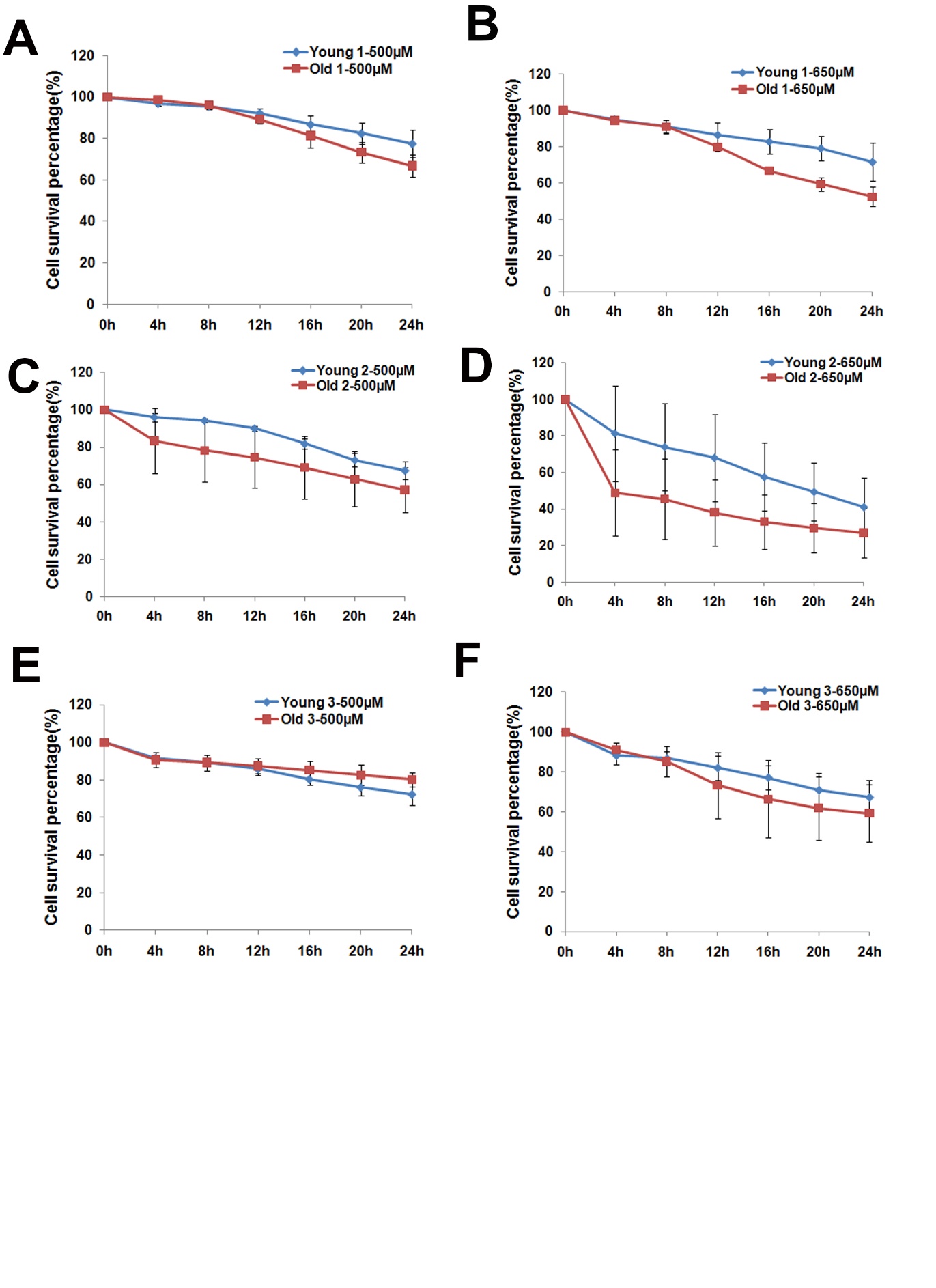


**Figure S1** Young and old hMDSCs exhibit similar oxidative stress resistance. Young and old hMDSCs were cultivated in media containing 500 and 650 µM H_2_O_2_ for 24 hrs in the presence of PI (which labels dead cells) and imaged using a live imaging system. The number of surviving cells and cell survival rate was quantified. (**A** and **B**) Comparison of cell survival of the young 1 and old 1 donor cells at 500 µM and 650 µM H_2_O_2_ respectively. (**C** and **D**) Comparison of cell survival of the young 2 and old 2 donor cells at 500 and 650 µM H_2_O_2_ respectively. (**E** and **F**) Comparison of cell survival of the young 3 and old 3 donor cells at 500 µM and 650 µM H_2_O_2_ respectively. No differences were observed at either concentrations for young and old donors. Notably, all hMDSCs were highly resistant to oxidative stress using H_2_O_2_, with survival rates at 650 µM being >50% for most of the cell populations tested.

Table S1: primers information:

| Gene Name | Accession # | Primer (5' to 3') | Product size  (bp) | Annealing temperature |
| --- | --- | --- | --- | --- |
| SOX9 | [NM_000346.3](http://www.ncbi.nlm.nih.gov/nucleotide/182765453?report=genbank&log$=nucltop&blast_rank=28&RID=P5NRB0NG01S) | F: GCTCAGCAAGACGCTGGGCA | 249 | 55 |
|  |  | R: CCGGAGGAGGAGTGTGGCGA |  |  |
| OSX | AF477981.1 | F: GCAGCTAGAAGGGAGTGGTG | 212 | 55 |
|  |  | R: AAGCCTTGCCATACACCTTG |  |  |
| RUNX2 | BC108920.1 | F: GGTACCAGATGGGACTGTGG | 111 | 55 |
|  |  | R: TCGTTGAACCTTGCTACTTGG |  |  |
| COX2 | [NM_000963.2](http://www.ncbi.nlm.nih.gov/nucleotide/223941909?report=genbank&log$=nucltop&blast_rank=6&RID=P5MN1M8E01S) | F:GCGAGGGCCAGCTTTCACCA | 225 | 55 |
|  |  | R: CCTGCCCCACAGCAAACCGT |  |  |
| IGF1 | M29644.1 | F: TGGATGCTCTTCAGTTCGT | 265 | 55 |
|  |  | R: CCTGCACTCCCTCTACTTGC |  |  |
| IGF2 | BC000531.1 | F: ATGACACCTGGAAGCAGTCC | 149 | 55 |
|  |  | R: GTCTTGGGTGGGTAGAGCAA |  |  |
| GPX1 | M21304.1 | F:CTCTTCGAGAAGTGCGAGGT | 236 |  |
|  |  | R:TCGATGTCAATGGTCTGGAA |  |  |
| GAPDH | BC083511.1 | F: GCCTTCCGTGTCCCCACTGC | 211 | 55 |
|  |  | R: CAATGCCAGCCCCAGCGTCA |  |  |

F: Forward; R: reverse
